# Marinacci Communication With Recovery of the Thumb, Finger, and Wrist Flexion After a High Median Nerve Injury: A Case Report and Review of Literature

**DOI:** 10.7759/cureus.34671

**Published:** 2023-02-06

**Authors:** Harvey Chim, Ramin Shekouhi, Rachel Cohen-Shohet

**Affiliations:** 1 Plastic and Reconstructive Surgery, University of Florida College of Medicine, Gainesville, USA

**Keywords:** median nerve variation in its formation and course, variation, hand trauma, anatomy, ulnar nerve, marinacci, median nerve

## Abstract

The Marinacci communication (MC) contains fibers from the ulnar to the median nerve in the forearm in a proximal to distal fashion. This rare ulnar-to-median nerve anomalous communication has mainly been reported as an incidental finding. In the case presented here, this anatomical variation led to rapid recovery of the thumb, finger, and wrist flexion following a high above elbow complete median nerve injury. A 17-year-old female was involved in an all-terrain vehicle rollover accident and had her right elbow crushed. She presented with no motor or sensory function in the forearm and hand, with a weak monophasic radial artery signal and no palpable pulse. She underwent surgery and was treated with 12 cm interposition cable sural nerve grafting. Although recovery after a high median nerve injury is often prolonged and incomplete, the MC resulted in the recovery of sensation and motor function through muscles typically innervated by the median nerve, following a complete high median nerve injury. In the presence of anomalous recovery following median nerve injury or unusual electrophysiological findings, an MC should be considered as a cause.

## Introduction

Recovery after a high median nerve injury is often prolonged and incomplete with a poor prognosis. The Marinacci communication (MC) contains fibers from the ulnar to the median nerve in the forearm in a proximal-to-distal fashion [1-3]. It was first described in 1964 in a patient who had trauma to the median nerve in the forearm with subsequent loss of function of forearm flexor muscles but retained function of median nerve innervated hand muscles [1]. Another case was reported as an unexpected anatomical finding during routine carpal tunnel release [2].

Due to the paucity of clinical cases, the exact incidence of the MC is unknown. An electrophysiological study of normal volunteers reported the prevalence in the general population as 3.5%. In addition, the clinical ramifications of different patterns of MC are unknown due to its rarity. Here, we report a case of a high median nerve injury with accelerated recovery of the wrist, thumb, and finger flexors, consistent with a proximal MC.

## Case presentation

A 17-year-old female was involved in an all-terrain vehicle (ATV) rollover accident and had her right elbow crushed by the ATV handlebar. She presented with multiple injuries to the right upper extremity. These included brachial artery transection, median nerve transection with a long gap, distal biceps avulsion, flexor pronator mass avulsion off the distal humerus, elbow ulnar collateral ligament avulsion with missing medial epicondyle, and a large soft-tissue defect measuring 20 × 16 cm.

**Figure 1:**
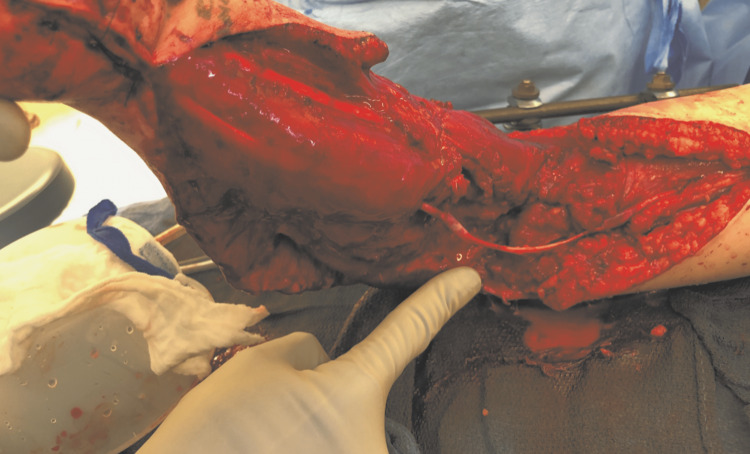
Segmental defect of the median nerve extending proximal to the elbow repaired with a 12 cm interposition cable sural nerve graft (indicated by the finger).

She presented with no motor or sensory function in the forearm and hand, with a weak monophasic radial artery signal and no palpable pulse. She was taken to the operating room urgently by the vascular surgery team and underwent reconstruction with a brachial-to-brachial artery great saphenous vein bypass. The orthopedic trauma team repaired the elbow ulnar collateral ligament, and a multiplanar external fixator was placed for stabilization of the elbow. The ulnar nerve was found to be in continuity but there was a segmental gap of the median nerve, which was repaired with a 12 cm cable sural nerve graft by the hand surgery team (Figure 1). This was followed by coverage with a pedicled abdominal flap, which was subsequently divided in a staged fashion for coverage of the soft-tissue defect, together with skin grafts.

Weak recovery of finger extension and some sensation in the radial nerve distribution was noted three months postoperatively. Four months postoperatively, finger extension had strengthened to Medical Research Council (MRC) Grade 3/5. Five months postoperatively, she reported trace finger flexion. At seven months postoperatively, she was noted to have MRC Grade 3 flexor pollicis longus (FPL), Grade 3 flexor digitorum profundus (FDP) to all fingers, Grade 4 flexor digitorum superficialis (FDS) to all fingers, Grade 3 flexor carpi radialis (FCR) and palmaris longus (PL), Grade 4 flexor carpi ulnaris (FCU), Grade 3 intrinsic muscle strength, and Grade 4+ wrist and finger extension. She also had recovering sensation over the ring and small fingers but not in the median nerve distribution in the hand. Tinel’s over the median nerve was noted 5 cm proximal to the elbow.

**Figure 2:**
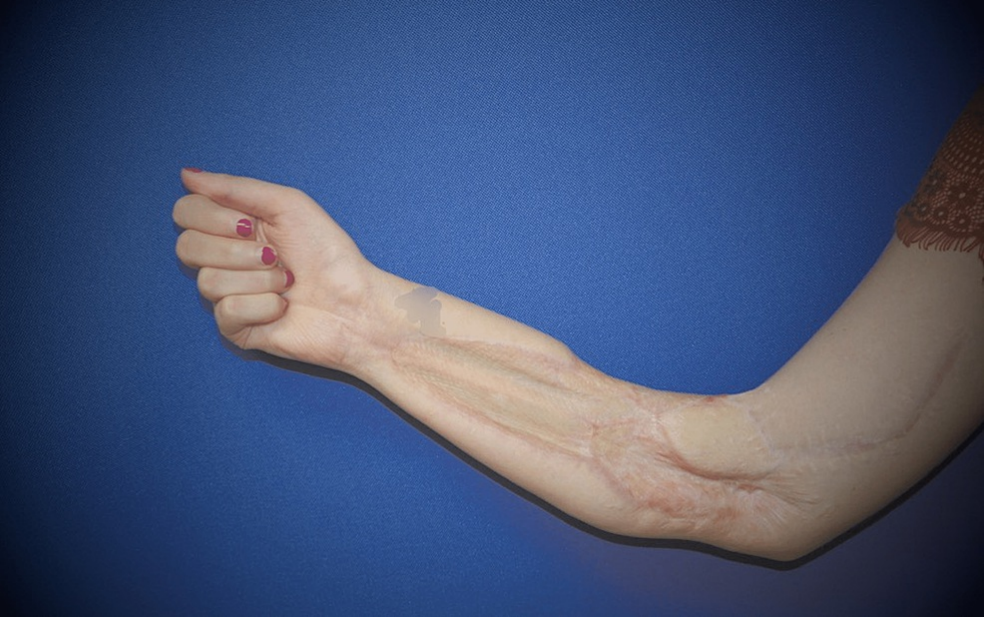
One year postoperatively, the patient is able to make a full fist.

**Figure 3:**
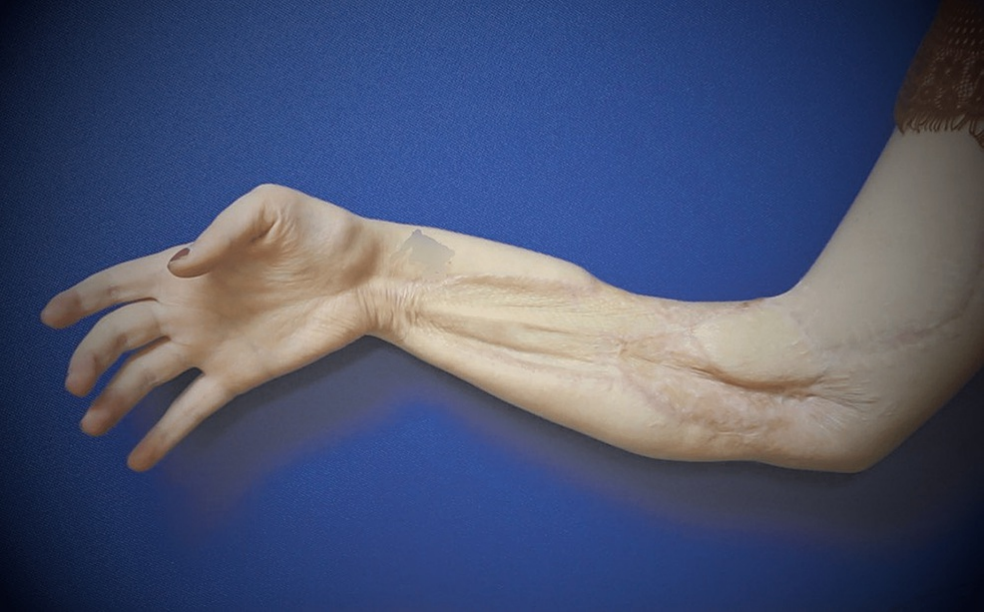
One year postoperatively, the patient is able to flex the thumb.

At 11 months postoperatively, FCR and PL had strengthened to Grade 4. However, she still noted that flexion in the thumb, index, and long fingers was somewhat weaker than the ring and small fingers. There was no recovery of sensation in the median nerve distribution in the hand, nor recovery of thumb opposition and abduction. Tinel’s was noted 2 cm proximal to the elbow, still over the proximal aspect of the cable sural nerve graft. She was able to make a full fist (Figure 2). In addition, she had strong thumb flexion through the FPL (Figure 3). The range of motion in the thumb interphalangeal joint was limited due to lack of use. Video 1 shows the good function of the hand. Unfortunately, the patient did not return for further follow-up visits.

**Video 1:** Hand function at one year postoperatively.

Electromyogram (EMG) performed four months postoperatively did not show any recovery in the form of motor unit potentials (MUPs) or recruitment in the median and ulnar nerve innervated muscles. Follow-up EMG one year postoperatively showed MUPs in the ulnar nerve innervated muscles. MUPs were also noted in the FDP but the FPL could not be assessed due to thick scar tissue in the volar forearm. No evidence of recovery was found in the abductor pollicis brevis (APB).

## Discussion

High median nerve injuries, considered those above the elbow, are uncommon, with a reported incidence of 0.1% of 1,385 injuries to nerves in the upper extremity [4]. Paralysis of flexion involving the thumb interphalangeal joint and the proximal and distal interphalangeal joints of the index finger is always found following a high median nerve injury [5]. Due to the poor prognosis for recovery following direct repair or nerve grafting, nerve or tendon transfers are commonly performed to increase the chance of a meaningful recovery [6].

This patient initially had complete paralysis of the right upper extremity. Recovery of the ulnar and radial nerves innervated muscles eventually occurred as these nerves were in continuity. Recovery of function in median nerve innervated muscles with a 12 cm interposition cable sural nerve graft to a median nerve injury proximal to the elbow would typically be expected to occur after at least 12 to 18 months, with a significant chance of incomplete or no recovery. Interestingly, recovery of flexion of the thumb, index, and long fingers occurred at the same time as the recovery of ulnar nerve innervated muscles by seven months post-reconstruction, supporting the hypothesis that function to these muscles occurred via an MC from the ulnar nerve.

This rare ulnar-median anomalous communication has mainly been reported as an incidental finding and the majority of patients diagnosed with am MC had noncontributory symptoms (Table 1) [7]. Accordingly, only two studies reported upper extremity neurologic manifestations that resulted in the diagnosis of MC [2,8]. Notably, both of these patients had a previous history of upper extremity trauma. Hence, it is speculated that the prevalence of MC has been underreported. A review of the literature suggests that this normal anatomic variation tends to often occur bilaterally [8,9]. Being aware of the presence of an anomalous nerve communication is of great importance when performing surgery as it increases the risk for iatrogenic nerve damage [2]. Findings suggestive of MC on EMG include conduction block on median nerve stimulation and, on ulnar nerve stimulation, increased compound muscle action potential (CMAP) when stimulated proximally compared to distally [3].

**Table 1:** Review of the literature of the Marinacci communication. ADM = abductor digiti minimi; APB = abductor policies brevis; CMAP = compound muscle action potential; FDP = flexor digiti minimi; MUP = motor unit potential; NCD = nerve conduction studies; RTI = road traffic injury

Author/year	N (F; M)	Mean age (years)	Side	Symptoms	Symptom duration	Cause of symptoms	Motor weakness	NCD median nerve	NCD ulnar nerve
Stancić et al., 2000 [2]	1 (0, 1)	36	Unilateral	Pain and hypoesthesia from the right thumb to the ring finger	Three months	Previous Colles’ fracture	No	Low sensory nerve conduction velocity, low CMAP amplitude in the APB muscle both distally and proximally, and lower APB CMAP compared with ulnar stimulation	N/A
Meenakshi-Sundaram et al., 2003 [3]	4 (N/A)	N/A	Bilateral in three patients	Low back pain in three patients, one patient R/O masculopathy	N/A	N/A	No	Proximal amplitude reduction than distally	Lesser ADM CMAP distally, and decreased APB CMAP distally
Nimma and Bhat, 2020 [9]	1 (1; 0)	47	Bilateral	Left hemibody hypoesthesia, lower extremity radicular pain	N/A	Unknown	No	Increased APB CMAP distally, and normal distal latency and velocity	Increased APB CMAP proximally
Chang et al., 2021 [7]	1 (0; 1)	56	Unilateral	Neck pain, and hypoesthesia of the left shoulder	Three weeks	C5-C6 radiculopathy	No	Proximal conduction block in APB, and higher proximal APB amplitude than distally	Increased distal latency and decreased velocity in sensation, and normal motor study
Wakode and Ravi, 2021 [8]	1 (1; 0)	Mid 20s	Bilateral	Lack of flexion in the right thumb and index fingers, and hypoesthesia of the right second, third, and fourth fingers	Three weeks	RTI	Yes	Proximal conduction block in APB, and normal conduction with distal stimulation	Decreased amplitudes at the ADM with proximal and distal ulnar stimulation, and normal APB CMAP with proximal stimulation
Our case	1 (1; 0)	17	Unilateral	No motor or sensory function in the forearm and hand	N/A	RTI	Yes	MUPs were also noted in the FDP	MUPs in the ulnar nerve innervated muscles

In our case, EMG provided limited information as some muscles could not be assessed due to thick scar tissue in the volar forearm. Unfortunately, direct visualization of the communication was not possible due to the extent of her injuries. Nevertheless, to our knowledge, this is the first report of an MC resulting in the recovery of the thumb, finger, and wrist flexion through muscles typically innervated by the median nerve, following a complete high median nerve injury.

## Conclusions

High median nerve injuries, considered those above the elbow, are uncommon and the recovery is often prolonged and incomplete. In this case, however, MC led to the accelerated recovery of thumb, finger, and wrist flexion following a high above elbow complete median nerve injury. The MC is considered a normal anatomical variation of the nerve anastomosis and should be taken into consideration during surgical procedures in the upper extremity.
